# Immunogenicity and safety study of 23-valent pneumococcal polysaccharide vaccine revaccination among elderly individuals aged 60–70 years in Shanghai, China

**DOI:** 10.3389/fimmu.2025.1623611

**Published:** 2025-07-17

**Authors:** Jing Qiu, Zhi Li, Fang Huang, Zhuoying Huang, Xiufang Liang, Juan Li, Yuting Liao, Xiang Guo, Xiaodong Sun

**Affiliations:** ^1^ Department of Immunization, Shanghai Municipal Center for Disease Control and Prevention, Shanghai, China; ^2^ Department of Immunization, Yangpu District Center for Disease Control and Prevention, Shanghai, China

**Keywords:** elderly, revaccination, 23-valent pneumococcal polysaccharide vaccine, immunogenicity, safety

## Abstract

**Background:**

To understand the immunogenicity and safety of the 23-valent pneumococcal polysaccharide vaccine in elderly individuals aged 60–70 years in Shanghai after revaccination.

**Methods:**

A total of 330 elderly people aged 60–70 years were recruited to study the immunogenicity and safety of PPSV23 revaccination. The group with a history of PPSV23 vaccination and an interval of 5 years or more was selected as the revaccination group (n=220), and the group without any pneumococcal vaccine was selected as the first vaccination group (n=110).

**Results:**

In terms of immunogenicity, the GMCs of all serotypes before and after immunization were 1.15-21.57 µg/ml and 1.62-33.19 µg/ml, respectively, in the revaccination group, and the GMCs of all serotypes after immunization were higher than those before immunization (*P*<0.0001). After immunization, the total GMI of antibodies in the revaccination group was lower than that in the first vaccination group [1.62(1.57, 1.67) vs 3.20(2.82, 3.57), *P*<0.0001], and the GMIs of all serotypes in the revaccination group were also lower than those in the first vaccination group [(1.40-1.92) vs (1.82-4.69), *P*<0.0001]. In terms of safety, no serious adverse events occurred during the study and all adverse events that occurred were mild or self-limiting. From 0–30 days after immunization, 7 patients in the revaccination group and 14 patients in the first vaccination group experienced adverse events, with incidence rates of 3.18% and 12.73%, respectively, which were lower in the revaccination group than in the first vaccination group (*P*=0.0008).

**Conclusions:**

Compared with those before vaccination, the antibody levels of elderly people aged 60–70 years in Shanghai who were inoculated with PPSV23 for 5 years or more tended to increase but were lower than those in the first vaccination group. The safety of revaccination with PPSV23 was favorable.

**Clinical Trial Registration:**

http://www.chictr.org.cn, identifier ChiCTR2100042000; http://clinicaltrials.gov, identifier NCT04701788.

## Introduction

*Streptococcus pneumoniae* (*Spn*), abbreviated as pneumococcus, is a common conditionally pathogenic bacterium that can invade different parts of the body, leading to a series of diseases, such as otitis media, sinusitis, pneumonia, meningitis, bacteremia, etc., and pneumococcal disease (PD) is a serious public health problem worldwide (1, 2). According to the World Health Organization (WHO), about 75% of cases of invasive pneumococcal disease (IPD) occur in children aged <2 years, and case fatality rates from IPD in children can be high, ranging up to 20% for septicaemia and 50% for meningitis in low- and middle-income countries (3). A systematic analysis of the global burden of disease revealed that *Spn* was the leading cause of lower respiratory infection morbidity and mortality globally, contributing to more deaths than all other etiologies combined in 2016 (4). In China, the disease burden of PD is equally severe (5–8). About 2.5 million people suffer from PD every year, resulting in 125,000 deaths, mainly among middle-aged and elderly people over 50 years old and infants under 1 year old with relatively weak immunity (9).

Vaccination is one of the important and effective means for the prevention and control of PD. As early as 2008, the WHO classified PD as a disease requiring “very high priority” for vaccine prevention (10). The 23-valent pneumococcal polysaccharide vaccine (PPSV23) has been proven to be an effective means of preventing *Spn* infections, particularly for IPD (11). The application of PPSV23 in the elderly population can effectively reduce the incidence of community-acquired pneumonia and prevent diseases associated with *Spn* infection (12, 13). In China, PPSV23 produced by Chengdu Institute of Biological Products Limited Liability Company contains serotypes 1, 2, 3, 4, 5, 6B, 7F, 8, 9N, 9V, 10A, 11A, 12F, 14, 15B, 17F, 18C, 19A, 19F, 20, 22F, 23F And 33F. It was approved in Chinese Mainland in 2006 for susceptible people aged 2 years and above. Shanghai has fully implemented free vaccination with PPSV23 for registered elderly people aged 60 and above since 2013, and the implementation results have shown excellent vaccination safety and health economics (14, 15).

Some studies have shown that the effectiveness of PPSV23 for preventing IPD in the elderly population decreases with time. An ecological study conducted by Andrews et al. in England and Wales showed that the protective effect of PPSV23 against serotype specific IPD ranged from 48% (95% CI: 32-60%) within 2 years to 15% (95% CI: -3% -30%) after 5 years (16). Another study conducted by MANOFF et al. in the United States involving 1,008 subjects showed that the functional antibody levels of people over 65 years old decreased over time (17). The research by SHAPIROED et al. showed that the effect of PPSV23 vaccination on IPD is the best occurring at <3 years after vaccination and the worst results occurring at >5 years (18). There is still controversy over whether it is necessary to revaccinate the elderly population with PPSV23. The American Advisory Committee on Immunization Practices (ACIP) and the 2021 guidelines from the Japanese Ministry of Health, Labour and Welfare both recommend that healthy elderly individuals receive a single dose of PPSV23, while high-risk populations such as those without spleens or immunosuppressants are recommended to receive PPSV23 again (19, 20). The Chinese expert consensus on pneumococcal vaccination for PPSV23 revaccination recommends that, for those who need to be revaccinated, the vaccination should be administered according to the instructions, and the revaccination interval should be at least 5 years (2). To further validate the safety and efficacy of revaccination of domestically produced PPSV23 in elderly individuals, the population-based observational studies were conducted simultaneously in Chengdu (21) and Shanghai, China. By the end of 2023, more than 1.83 million elderly people had been vaccinated with PPSV23 free of charge in Shanghai, and approximately 3% of them had been revaccinated. In this study, we investigated the immunogenicity and safety of revaccination with PPSV23 in elderly people aged 60–70 years in Shanghai and provided a scientific basis for improving the immunization strategy for elderly people vaccinated with PPSV23.

## Methods

### Study design and participants

This single-center, controlled clinical study was conducted by the Shanghai Municipal Center for Disease Control and Prevention in Changning, Hongkou, Jiading and Qingpu districts of Shanghai, China, from March to August 2021. A total of 330 elderly people aged 60–70 years were recruited to study the immunogenicity and safety of PPSV23 revaccination. The group with a history of PPSV23 vaccination and an interval of 5 years or more was selected as the revaccination group (n=220), and the group without any pneumococcal vaccine was selected as the first vaccination group (n=110). Subjects who are allergic to PPSV23 vaccine components, have immunodeficiency, are under treatment for malignant tumors, have received nonspecific immunoglobulin injections within 3 months prior to enrollment, have a body temperature >37.0°C, have coagulation disorders, and those the researchers believe may influence the evaluation of the trial will be excluded.

### Research process and research content

After the study subjects were enrolled, their general information was collected by uniformly trained staff. With informed consent, 5ml of venous blood was collected before immunization and the serum was separated and divided into two tubes, with a minimum serum volume of 0.5ml in each tube. The tubes were stored at a temperature below -20°C. Then, one dose of PPSV23 was injected subcutaneous or intramuscularly into the lateral deltoid muscle of the upper arm according to the instruction. After an interval of 30 days, blood samples were collected again using the same method. The blood collection window period was -2 to 10 days.

For laboratory testing, an enzyme-linked immunosorbent assay (ELISA) was used to quantitatively detect 23 immunoglobulin G (IgG) antibodies against specific serotypes of *Spn* intercalated polysaccharide in human sera and to evaluate the geometric mean concentration (GMC), geometric mean increase (GMI) and 2-fold increase rate of the antibodies in human sera before and after inoculation with PPSV23.

For safety, the study subjects were required to stay for 30 minutes after one dose of PPSV23, and the staff issued diary cards for them to collect the occurrence of adverse events within 30 days after vaccination, in which the adverse events included localized solicited adverse events, systemic solicited adverse events, and non-solicited adverse events.

### Study vaccine

The vaccine administered in this study was PPSV23 produced by the Chengdu Institute of Biological Products Limited Liability Company, lot number 2020311, specification 0.5 ml/vial.

### Data sets

This study involved two types of datasets. The immunogenicity evaluation analysis used the immunogenicity Per-Protocol Set (PPS), that is, the subjects who met the inclusion/exclusion criteria, underwent full follow-up as required by the protocol, and were not excluded during the blind verification of serum results. The safety evaluation analysis used the Safety Set (SS), which included the study subjects who received the vaccine and underwent at least one safety evaluation.

### Statistical analysis

The data were statistically analyzed via SAS 9.4 software. The quantitative data were presented as x ± s, and the qualitative data were presented as frequencies. The chi-square test was used to compare the mean 2-fold increase rates of antibodies between groups, and the paired t test was used to compare the GMCs and GMIs of antibodies before and after immunization in the same group. Local and systemic reactions after vaccination were statistically analyzed in all study subjects, and the incidence was compared between groups via the chi-square test. Two-sided test with test level α=0.05.

### Ethics

This study was approved by the institutional review board of the Shanghai Municipal Center for Disease Control and Prevention (No. 2020-96). This study was registered in January 2021 on a foreign website (http://clinicaltrials.gov, NCT04701788) and on a domestic website (http://www.chictr.org.cn, ChiCTR2100042000).

## Results

### Basic situation

**Figure 1:**
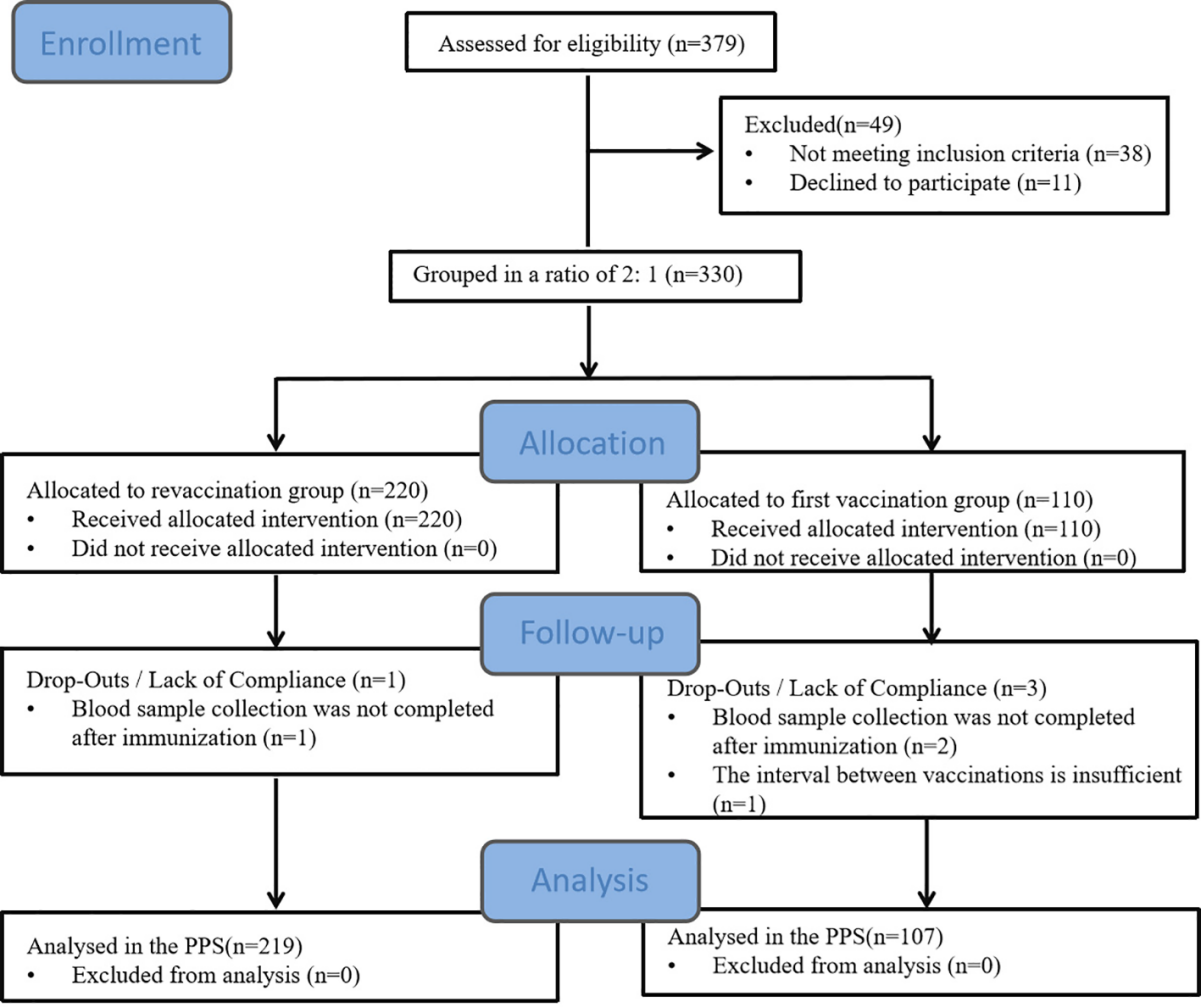
Study flow diagram of participants through the study period.

A total of 330 subjects were enrolled in this study, including 220 in the revaccination group and 110 in the first vaccination group. Four subjects were dislodged during the period, 326 subjects were included in the PPS, and 330 subjects were included in the SS. See **Table 1**; **Figure 1**.

**Table 1:** Basic characteristics of the study population.

Variant	PPS			SS		
	Revaccination group (n=219)	First vaccination group (n=107)	*P*	Revaccination group (n=220)	First vaccination group (n=110)	*P*
Age (years)	66.73 ± 1.23	64.59 ± 2.28	<0.0001	66.73 ± 1.22	64.69 ± 2.26	<0.0001
Sex (n,%)						
Male	131 (59.82%)	50 (46.73%)	0.0256	131 (59.55%)	51 (46.36%)	0.0232
Female	88 (40.18%)	57 (53.27%)		89 (40.45%)	59 (53.64%)	

### Immunogenicity

#### GMC levels

In the revaccination group, the GMCs of the 23 serotypes of *Spn* capsular polysaccharide IgG antibodies before and after immunization were 1.15-21.57 µg/ml and 1.62-33.19 µg/ml, respectively, and the level of the GMC of each type after immunization was higher than that before immunization, and the difference was statistically significant in all cases (*P*<0.0001). Similarly, in the first vaccination group, the GMCs of all serotypes were 0.60-10.84 µg/ml and 1.59-31.30 µg/ml before and after immunization, respectively, and the level of each type after immunization was significantly higher than that before immunization (*P*<0.0001). In addition, GMCs of all serotypes in the revaccination group before immunization were higher than those in the first vaccination group (*P*<0.0001), and GMC of type 22F in the revaccination group after immunization was higher than that in the first vaccination group [9.61(8.87, 10.41) vs 7.43(6.35, 8.71), *P*=0.0033]. See **Table 2**.

**Table 2:** GMCs of IgG antibodies against 23 serotypes in both groups.

Type		Revaccination group (n=219)			First vaccination group (n=107)			*P* ^3^
		Total	GMC (ug/ml, 95%CI)	*P* ^1^	Total	GMC (ug/ml, 95%CI)	*P* ^2^	
Pn1	pre-immunization	219	5.65 (5.24, 6.09)	<0.0001	107	3.58 (3.23, 3.97)	<0.0001	<0.0001
	post-immunization	219	9.38 (8.64, 10.19)		107	10.26 (8.70, 12.11)		0.2788
Pn2	pre-immunization	219	6.57 (6.03, 7.15)	<0.0001	107	3.34 (2.91, 3.84)	<0.0001	<0.0001
	post-immunization	219	10.65 (9.76, 11.62)		107	10.46 (8.55, 12.79)		0.8480
Pn3	pre-immunization	219	1.15 (1.07, 1.24)	<0.0001	107	0.87 (0.78, 0.97)	<0.0001	<0.0001
	post-immunization	219	1.62 (1.50, 1.74)		107	1.59 (1.40, 1.81)		0.9341
Pn4	pre-immunization	219	2.59 (2.38, 2.82)	<0.0001	107	1.35 (1.20, 1.53)	<0.0001	<0.0001
	post-immunization	219	4.02 (3.66, 4.41)		107	3.65 (3.02, 4.41)		0.3694
Pn5	pre-immunization	219	4.52 (4.12, 4.95)	<0.0001	107	2.62 (2.32, 2.96)	<0.0001	<0.0001
	post-immunization	219	7.04 (6.45, 7.69)		107	6.46 (5.41, 7.71)		0.3799
Pn6B	pre-immunization	219	5.62 (5.14, 6.14)	<0.0001	107	2.98 (2.62, 3.38)	<0.0001	<0.0001
	post-immunization	219	9.21 (8,45, 10.04)		107	8.94 (7.43, 10.77)		0.8303
Pn7F	pre-immunization	219	6.12 (5.57, 6.73)	<0.0001	107	3.03 (2.62, 3.50)	<0.0001	<0.0001
	post-immunization	219	10.45 (9.53, 11.45)		107	11.33 (9.39, 13.67)		0.4789
Pn8	pre-immunization	219	9.32 (8.56, 10.16)	<0.0001	107	4.43 (3.92, 5.00)	<0.0001	<0.0001
	post-immunization	219	16.58 (15.25, 18.03)		107	14.50 (12.20, 17.22)		0.1581
Pn9N	pre-immunization	219	8.53 (7.78, 9.35)	<0.0001	107	3.56 (3.08, 4.10)	<0.0001	<0.0001
	post-immunization	219	13.91 (12.81, 15.10)		107	16.51 (13.43, 20.31)		0.1235
Pn9V	pre-immunization	219	5.11 (4.65, 5.62)	<0.0001	107	2.19 (1.89, 2.54)	<0.0001	<0.0001
	post-immunization	219	8.65 (7.92, 9.46)		107	8.25 (6.85, 9.95)		0.6276
Pn10A	pre-immunization	219	9.94 (9.13, 10.81)	<0.0001	107	6.14 (5.47, 6.89)	<0.0001	<0.0001
	post-immunization	219	16.97 (15.50, 18.57)		107	18.92 (15.79, 22.66)		0.2569
Pn11A	pre-immunization	219	4.84 (4.42, 5.30)	<0.0001	107	2.81 (2.44, 3.24)	<0.0001	<0.0001
	post-immunization	219	7.84 (7.17, 8.57)		107	8.48 (7.13, 10.08)		0.4349
Pn12F	pre-immunization	219	1.86 (1.72, 2.03)	<0.0001	107	1.22 (1.09, 1.36)	<0.0001	<0.0001
	post-immunization	219	2.70 (2.48, 2.94)		107	2.68 (2.30, 3.11)		0.8567
Pn14	pre-immunization	219	21.57 (19.69, 23.62)	<0.0001	107	10.84 (9.61, 12.22)	<0.0001	<0.0001
	post-immunization	219	33.19 (29.99, 36.74)		107	31.30 (26.26, 37.30)		0.4845
Pn15B	pre-immunization	219	15.55 (14.02, 17.24)	<0.0001	107	5.71 (4.95, 6.58)	<0.0001	<0.0001
	post-immunization	219	28.01 (25.50, 30.76)		107	26.75 (22.26, 32.13)		0.6801
Pn17F	pre-immunization	219	1.39 (1.26, 1.53)	<0.0001	107	0.60 (0.52, 0.68)	<0.0001	<0.0001
	post-immunization	219	2.27 (2.07, 2.49)		107	2.48 (2.05, 3.00)		0.4012
Pn18C	pre-immunization	219	5.61 (5.15, 6.10)	<0.0001	107	2.56 (2.24, 2.94)	<0.0001	<0.0001
	post-immunization	219	8.73 (8.05, 9.46)		107	8.80 (7.41, 10.45)		0.8624
Pn19A	pre-immunization	219	10.61 (9.67, 11.65)	<0.0001	107	5.79 (5.18, 6.48)	<0.0001	<0.0001
	post-immunization	219	16.25 (14.83, 17.81)		107	14.63 (12.22, 17.51)		0.2552
Pn19F	pre-immunization	219	8.64 (7.97, 9.37)	<0.0001	107	5.40 (4.81, 6.07)	<0.0001	<0.0001
	post-immunization	219	13.83 (12.70, 15.06)		107	14.57 (12.26, 17.31)		0.6601
Pn20	pre-immunization	219	8.71 (7.70, 9.84)	<0.0001	107	3.58 (3.08, 4.16)	<0.0001	<0.0001
	post-immunization	219	16.74 (14.81, 18.91)		107	16.54 (13.14, 20.82)		0.8673
Pn22F	pre-immunization	219	6.76 (6.26, 7.31)	<0.0001	107	3.73 (3.32, 4.18)	<0.0001	<0.0001
	post-immunization	219	9.61 (8.87, 10.41)		107	7.43 (6.35, 8.71)		0.0033
Pn23F	pre-immunization	219	3.96 (3.64, 4.31)	<0.0001	107	2.52 (2.22, 2.85)	<0.0001	<0.0001
	post-immunization	219	6.42 (5.86, 7.05)		107	5.93 (4.98, 7.05)		0.3802
Pn33F	pre-immunization	219	13.39 (12.11, 14.79)	<0.0001	107	5.70 (5.04, 6.43)	<0.0001	<0.0001
	post-immunization	219	21.37 (19.53, 23.39)		107	25.55 (21.29, 30.66)		0.0717

*P* ^1^ represents for the comparison of GMCs before and after immunization in the revaccination group, the *P* ^2^ represents for the comparison of GMCs before and after immunization in the first vaccination group, and the *P* ^3^ represents for the comparison of GMCs between the revaccination group and the first vaccination group.

#### GMI levels

The total antibody GMI of the revaccination group and the first vaccination group was 1.62(1.57, 1.67) and 3.20(2.82, 3.57), respectively, and that of the revaccination group was lower than that of the first vaccination group (*P*<0.0001). The GMIs of the 23 serotypes of antibodies in the two groups were 1.40-1.92 and 1.82-4.69, respectively, and the GMI of the antibodies of each serotype in the revaccination group was lower than that in the first vaccination group (*P*<0.0001). See **Table 3**.

**Table 3:** GMIs of IgG antibodies against 23 serotypes in both groups.

Type	Revaccination group (n=219)	First vaccination group (n=107)	*P*
	GMI (95%CI)	GMI (95%CI)	
Pn1	1.66 (1.58,1.75)	2.87 (2.48,3.32)	<0.0001
Pn2	1.62 (1.54,1.71)	3.13 (2.63,3.73)	<0.0001
Pn3	1.40 (1.35,1.45)	1.82 (1.63,2.03)	<0.0001
Pn4	1.55 (1.47,1.64)	2.70 (2.28,3.19)	<0.0001
Pn5	1.56 (1.48,1.64)	2.46 (2.11,2.88)	<0.0001
Pn6B	1.64 (1.56,1.72)	3.01 (2.58,3.50)	<0.0001
Pn7F	1.71 (1.61,1.81)	3.74 (3.18,4.40)	<0.0001
Pn8	1.78 (1.69,1.88)	3.27 (2.80,3.83)	<0.0001
Pn9N	1.63 (1.54,1.73)	4.64 (3.79,5.69)	<0.0001
Pn9V	1.69 (1.60,1.79)	3.76 (3.20,4.43)	<0.0001
Pn10A	1.71 (1.62,1.80)	3.08 (2.62,3.62)	<0.0001
Pn11A	1.62 (1.54,1.70)	3.02 (2.55,3.56)	<0.0001
Pn12F	1.45 (1.38,1.51)	2.19 (1.95,2.47)	<0.0001
Pn14	1.54 (1.44,1.64)	2.89 (2.44,3.42)	<0.0001
Pn15B	1.80 (1.68,1.93)	4.69 (3.87,5.68)	<0.0001
Pn17F	1.63 (1.53,1.74)	4.16 (3.46,4.99)	<0.0001
Pn18C	1.56 (1.48,1.64)	3.43 (2.91,4.04)	<0.0001
Pn19A	1.53 (1.46,1.61)	2.53 (2.17,2.95)	<0.0001
Pn19F	1.60 (1.52,1.68)	2.70 (2.35,3.09)	<0.0001
Pn20	1.92 (1.79,2.06)	4.62 (3.74,5.70)	<0.0001
Pn22F	1.42 (1.36,1.48)	2.00 (1.77,2.25)	<0.0001
Pn23F	1.62 (1.53,1.72)	2.35 (2.02,2.74)	<0.0001
Pn33F	1.60 (1.50,1.70)	4.49 (3.75,5.36)	<0.0001
Total	1.62 (1.57,1.67)	3.20 (2.82,3.57)	<0.0001

### Mean 2-fold increase rates

**Figure 2:**
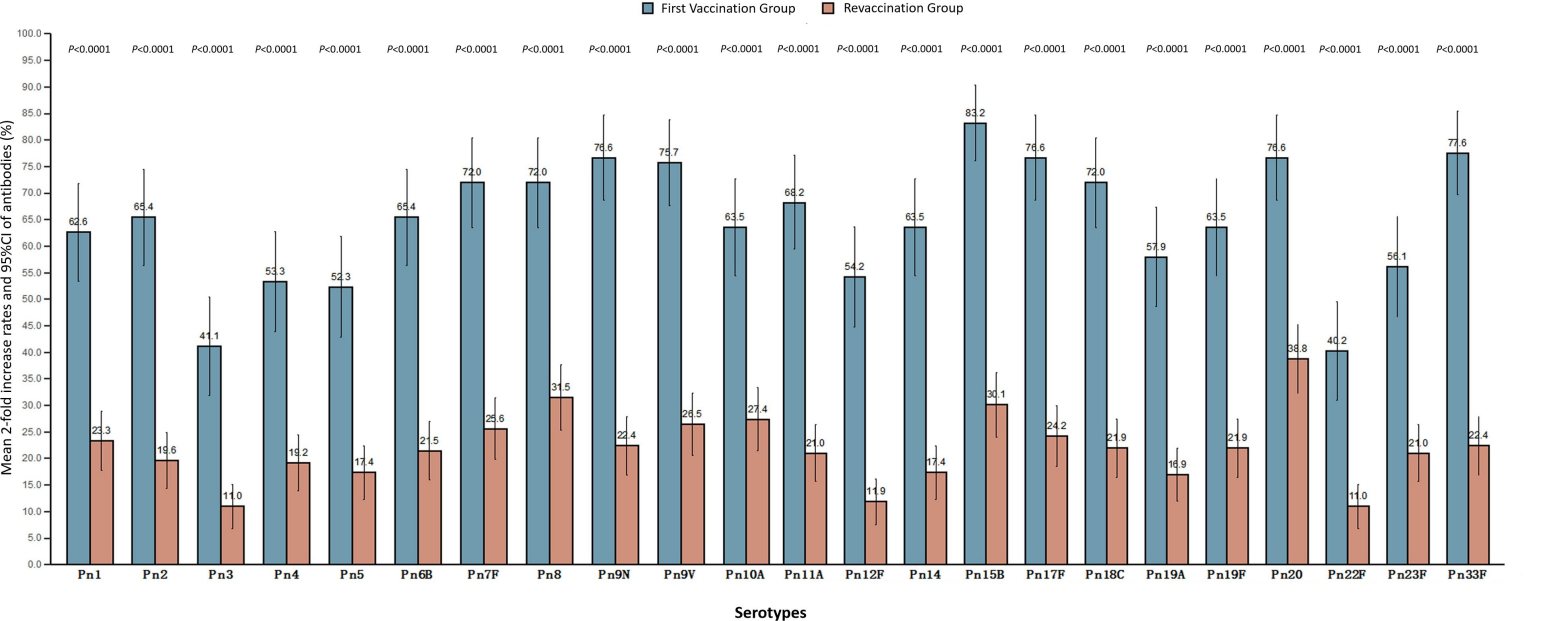
Mean 2-fold increase rates and 95%CI of IgG antibodies of 23 serotypes in both groups.

The mean 2-fold increase rate of antibodies in the revaccination group and the first vaccination group was 21.90% (19.07%, 24.72%) and 64.77% (59.80%, 69.74%), respectively, which was lower in the revaccination group than that in the first vaccination group (*P*<0.0001). The average 2-fold increase rates of antibodies to 23 serotypes in the two groups were 10.96%-38.81% and 40.19%-83.18%, respectively, and the average 2-fold increase rates of antibodies to all serotypes in the revaccination group were lower than those in the first-time vaccination group, and the differences were statistically significant (*P*<0.0001). See **Figure 2**.

### Safety

#### Occurrence of adverse events

From 0–30 days after PPSV23 vaccination, adverse events occurred in 7 patients in the revaccination group, with an adverse event rate of 3.18%, and 14 patients in the first vaccination group, with an adverse event rate of 12.73%. There was a statistically significant difference in incidence between two groups (*P* =0.0008), and the incidence in the revaccination group was lower than that in the first vaccination group. No serious adverse events occurred during the study observation period. See **Table 4**.

**Table 4:** Overall adverse events 0–30 days after vaccination in both groups.

Name of adverse events	Revaccination group(n=220)		First vaccination group(n=110)		*P*
	Number of occurrences/Number of individuals involved	Incidence rate (%)	Number of occurrences/Number of individuals involved	Incidence rate (%)	
Adverse events	13/7	3.18	25/14	12.73	0.0008
Solicited adverse events	8/3	1.36	17/11	10.00	0.0005
Unsolicited adverse events	5/4	1.82	8/5	4.55	0.1663
Serious adverse events	0/0	0.00	0/0	0.00	–

#### Analysis of symptoms of adverse events

From 0–30 days after PPSV23 vaccination, the number of localized symptomatic adverse events in the revaccination group and the first vaccination group was 2 (0.91%) and 7(6.36%), respectively, and the incidence rate in the revaccination group was lower than that in the first vaccination group (*P*=0.0075). The number of systemic solicited adverse events in the revaccination group and the first vaccination group was 2(0.91%) and 4(3.64%), respectively, and the difference in the incidence rates between two groups was not statistically significant (*P*=0.0981). There were no statistically significant differences in the occurrence of non-solicited adverse events between the two groups (*P*>0.05). See **Table 5**.

**Table 5:** Symptom analysis of adverse events 0–30 days after vaccination in both groups.

Adverse events classification	Revaccination group (n=220)		First vaccination group (n=110)		*P*
	Number of occurrences/Number of individuals involved	Incidence rate (%)	Number of occurrences/Number of individuals involved	Incidence rate (%)	
Solicited adverse events					
Local adverse events	6/2	0.91	11/7	6.36	0.0075
Ache	2/1	0.45	5/4	3.64	0.0440
Hard knot	0/0	0.00	2/1	0.91	0.3333
Redness	2/1	0.45	3/3	2.73	0.1098
Swollen	2/1	0.45	1/1	0.91	1.0000
Rash	0/0	0.00	0/0	0.00	–
Pruritus	0/0	0.00	0/0	0.00	–
Systemic adverse events	2/2	0.91	6/4	3.64	0.0981
Fever	2/2	0.91	1/1	0.91	1.0000
Stimulate or inhibit	0/0	0.00	0/0	0.00	–
Sickness	0/0	0.00	0/0	0.00	–
Anorexia	0/0	0.00	0/0	0.00	–
Diarrhea	0/0	0.00	0/0	0.00	–
Hypersomnia	0/0	0.00	3/2	1.82	0.1104
Acute allergic reaction	0/0	0.00	2/1	0.91	0.3333
Non-solicited Adverse events					
Infectious and infective diseases	2/1	0.45	0/0	0.00	1.0000
Skin and subcutaneous tissue diseases	0/0	0.00	0/0	0.00	–
Eye organ diseases	0/0	0.00	1/1	0.91	0.3333
Heart organ diseases	0/0	0.00	2/1	0.91	0.3333
Nervous system diseases	1/1	0.45	0/0	0.00	1.0000
Respiratory, thoracic, and mediastinal diseases	2/2	0.91	5/3	2.73	0.3380

## Discussion

The morbidity and mortality of PD and its associated complications are high in people aged 60 years and older, and pneumonia vaccination (including polysaccharide and conjugate vaccines) is one of the most cost-effective means of preventing *Spn* infection (22–24). A previous population–cohort study conducted in Shanghai suggested that most antibody levels of PPV23 can persist for more than 5 years (25). However, there is still controversy about the need to revaccinate the elderly population with PPSV23, and there have not been sufficient previous studies on PPSV23 revaccination in China. In this study, 330 elderly people aged 60–70 years were selected to conduct a clinical study on the immunogenicity and safety of PPSV23 revaccination in Shanghai. The results showed that the antibody GMC of elderly individuals in Shanghai increased to different degrees after 5 years of inoculation with PPSV23, and the safety of revaccination with PPSV23 was favorable. Thus, elderly people aged 60 years and older could consider revaccination with PPSV23 at least 5 years after the first vaccination, and since the PCV vaccine has been used in adults abroad with a good protective effect, we also look forward to introducing the PCV vaccine for sequential vaccination in the future.

About PPSV23 revaccination, Musher et al.’s study showed that both primary vaccination and revaccination with PPSV23 induce antibody responses that persist during 5 years of observation (26). Lackner et al.’s study also showed that revaccination with PPSV23 at least 5 years after primary vaccination is associated with a significant immune response for most of the serotypes tests in frail, chronically ill older nursing facility residents and revaccination was well tolerated (27). These are similar to the results of our study. In this study, before immunization, the antibody GMC levels of all serotypes in the revaccination group were higher than those in the first vaccination group, which was similar to the research results of Musher et al (26). And it suggested that protective antibodies were still present in the organism 5 years after PPSV23 vaccination. After immunization, in the revaccination group, except for the type 22F GMC level, which was higher than that in the first vaccination group [9.61 (8.87, 10.41) vs. 7.43 (6.35, 8.71), *P*=0.0033], the other antibody GMC levels were not significantly different between two groups, which was similar to the results of a study conducted by Kawakami (28) et al. in people >70 years old in Japan. That is, the antibody levels in the revaccination group and the first vaccination group were comparable after immunization. The reason why the GMC level of type 22F in the revaccination group was higher than that in the first vaccination group might be that type 22F could activate B cells more effectively, while other types were inhibited due to competition. However, the antibody GMI and mean 2-fold increase rate were lower in the revaccination group than those in the first vaccination group, possibly because the baseline levels of each antibody GMC in the revaccination group were higher than those in the first vaccination group, that is, the baseline levels of the two groups were inconsistent. However, it has also been shown (29) that although revaccination with PPSV23 induced an increase in the antibody GMC, it was still lower than the level after the first vaccination, which may be related to the depletion of the memory B-cell population (30).

In terms of safety, no serious adverse events occurred in the subjects during the study observation period in either the revaccination group or the first vaccination group, and those adverse events were usually mild or self-limiting, which is similar to the findings of previous studies (31). The incidence of localized adverse events was higher in the first vaccination group than that in the revaccination group in this study (0.91% vs 6.36%, *P*=0.0075), especially tenderness, which may be related to the fact that the subjects in the revaccination group were older and had a poorer sense of adverse events. It is also possible that during the first vaccination, the body had no pre-stored immunity to the pneumococcal polysaccharide antigen, and the immune system would initiate a stronger inflammatory response, resulting a higher incidence of local redness, swelling, pain and other reactions. However, the results of another study (32) revealed an increase in localized reactions (≥10.2cm) within 2 days after revaccination with PPSV23 compared with the first vaccination (RR=3.3, 95%CI: 2.1-5.1).

There are several shortcomings in this study. For instance, the sample size of this study is relatively small and stratified analyses of age, gender have not been conducted. Secondly, only ELISA for serum neutralizing antibodies was performed in this study, but functional antibody testing was not performed, nor was cellular immunity level testing. Also, the vaccination histories of herpes zoster vaccine, influenza vaccine and COVID-19 vaccine of the participants were not collected in this study. These are all the directions for subsequent in-depth research.

## Conclusion

The antibody levels of 60–70 year old people in Shanghai who were inoculated with PPSV23 for 5 years or more showed an increasing trend compared with those before vaccination, but were lower than those in the first vaccination group. The safety of revaccination with PPSV23 was favorable.

## Data Availability

The datasets presented in this article are not readily available because of contractual agreements with the sponsor (China Biotechnology Co., Ltd), which stipulated that the ownership of the raw data belonged to the sponsor. Requests to access the datasets should be directed to Haiping Chen from China Biotechnology Co., Ltd, chenhaiping@sinopharm.com.
